# Does habitat modification impact morphology, performance, and inflammatory responses in an amphibian with limited dispersal capacity (*Lisssotriton helveticus*)?

**DOI:** 10.1002/ece3.70114

**Published:** 2024-08-07

**Authors:** Soline Bettencourt‐Amarante, Robin Furet, Raphaëlle Abensur, Anthony Herrel

**Affiliations:** ^1^ UMR 7179 MECADEV CNRS/MNHN Paris France; ^2^ Department of Biology, Evolutionary Morphology of Vertebrates Ghent University Ghent Belgium; ^3^ Department of Biology University of Antwerp Wilrijk Belgium; ^4^ Naturhistorisches Museum Bern Bern Switzerland

**Keywords:** adaptation, amphibian, fragmentation, immunity, land‐use, locomotor performance, morphology

## Abstract

The environment of an organism exerts selective pressures that affect mobility, feeding, reproduction as well as predator–prey and conspecific interactions. Land use changes induced by human activities modify these selective pressures and may result in the adaptation of organisms. Amphibians are ectotherms that typically show a biphasic life cycle with an aquatic and terrestrial phase, which makes them particularly sensitive to environmental change. We studied the impact of habitat modifications on palmate newt populations in the Ile de France region across four types of habitats: urban, mixed, agricultural, and natural with at least two replicates for each habitat type. We measured the morphology of newts using callipers, quantified maximal running and swimming speed and acceleration using high‐speed video recordings, and quantified the swelling of the hind limb linked to an inflammatory reaction. Our results show that in urban habitats, newts are larger and heavier and have a better body condition. Females, moreover, have a larger head in natural habitats, possibly due to diet specialisation of females during the breeding season. In mixed and agricultural habitats, newts have longer limbs and show a tendency to run faster, possibly associated with the selective pressures on movement in mixed habitats. Differences in inflammatory responses were observed between sexes but not habitat types. Overall, our results show differences in morphology and trends for differences in performance in newts living in different habitats suggesting that animals are adapting to human‐induced changes in their environment.

## Graphical abstract

We studied the impact of habitat modifications on Palmate newt populations in the Ile de France region across four types of habitats: urban, mixed, agricultural, and natural. For each habitat, we measured the morphology of newts, quantified maximal running and swimming speed and acceleration, and quantified the immune response.

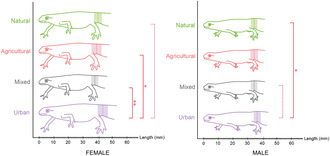

## 1 INTRODUCTION

Environmental variation and biotic interactions are the primary drivers of phenotypic variation. The physical features of the environment exert selective pressures on the ability of an organism to move, feed, and reproduce (Grenier et al., 2016). In addition, interactions with prey, predators, and conspecifics may impose selection on the phenotype of an organism (Adams & Rohlf, 2000; Liao et al., 2022; Losos, 2000). Organisms can respond to changes in their environment through selection on heritable variation or plastic responses. However, the ability of organisms to respond is dependent on the temporal nature of variation in the environment (Ghalambor et al., 2007). Anthropogenic modifications in the environment may lead to extremely rapid changes that may pose significant challenges to the ability of organisms to adapt (Hendry et al., 2008; Jacobs et al., 2019). Land use changes convert natural landscapes into modified environments due to human activity (Foley et al., 2005). These anthropogenic modifications impact the whole planet and its ecological functions (Steffen et al., 2007). It is generally accepted that the two main drivers of land‐use change are urbanisation and agriculture, which cause significant habitat fragmentation and deforestation and ultimately may cause biodiversity loss (DeFries et al., 2004), and this at an ever‐increasing rate.

Urbanisation and agriculture also modify landscape connectivity. Connectivity is defined as the extent to which individuals or gametes can move through a landscape (Taylor et al., 1993; Tischendorf & Fahrig, 2000). From a structural standpoint, habitat fragmentation generates patches of different sizes and compositions separated by barriers (Fahrig, 2003). This fragmentation obstructs species dispersal and gene flow (Hanski, 2015). From a functional standpoint, landscape connectivity impacts the ability of organisms to cross landscape obstacles depending on their behaviour and biological features (Betts et al., 2015; Pe'er et al., 2011; Trochet, Dechartre, et al., 2016; Trochet et al., 2019). Barriers to movement have consequences on ecological time scales (Cosgrove et al., 2018) and may impact the evolutionary trajectory of a population by selection on locomotor performance and the underlying limb morphology (Trochet et al., 2017). For instance, Trochet, Dechartre, et al. (2016) found short‐legged newts in the vicinity of roads unlike long‐legged newts which were observed in the vicinity of ponds in forested areas. In general, organisms with low mobility like amphibians are expected to be strongly impacted by changes in connectivity.

Amphibians, as ectotherms, are particularly vulnerable to environmental change. The IUCN lists 41% of all amphibians as threatened by extinction (IUCN, 2024). Most of the threats have an anthropogenic origin including drought, wildfire and extreme temperatures, habitat loss, degradation, fragmentation, introduction of invasive species, and disease (Blaustein et al., 2018; Burns et al., 2021; Nolan et al., 2023). Moreover, most amphibians have a complex biphasic life cycle. Eggs and tadpoles develop during an aquatic phase followed by metamorphosis. After metamorphosis, adults typically migrate to the terrestrial environment. Thus, amphibians need different types of habitats to complete their life cycle making them particularly vulnerable (Duellman & Trueb, 1994; Nolan et al., 2023). As poor dispersers, they have a high site fidelity (Blaustein et al., 1994; Duellman & Trueb, 1994) making them additionally highly sensitive to habitat modification.

In this study, we examined variation in morphology, locomotor performance, and inflammatory responses related to land use differences in an amphibian species with low mobility, the palmate newt, *Lissotriton helveticus*. We hypothesised that the degree of barriers to dispersal would impact morphology (Trochet, Le Chevalier, et al., 2016) and possibly locomotor performance. We predict that animals in urban or natural habitats confronted with significant barriers to dispersal would show longer limbs and greater locomotor performance (French et al., 2018; Winchell et al., 2018). We also explored whether variation in land use was reflected in immune responses and predict that animals from urban sites would show stronger inflammatory responses (French et al., 2008) because urban newts are likely subjected to greater stress and therefore their immune system would be challenged more often. Overall, we expect to see marked differences between animals in natural, agricultural, and urban habitats, with newts from mixed habitats being intermediate.

## 2 MATERIALS AND METHODS

### 2.1 Study species

This study focuses on the palmate newt (*Lissotriton helveticus*), a small‐sized species of newt (males: 50–80 mm; females: 55–95 mm; Duguet & Melki, 2003). Palmate newts have a complex biphasic life cycle, with adults spending most of the year on land and passing the reproductive season from February to July in an aquatic phase (Halliday, 1977). The species occupies a diverse range of stagnant or slow‐flowing water bodies (Duguet & Melki, 2003).

### 2.2 Habitat classification

We sampled 15 sites (four natural, six agricultural, three mixed, and two urban sites) in the Île‐de‐France region (France; see Table 1) during the breeding season. Newts generally show strong site fidelity and mostly move within a radius of less than 1 km (Phillips et al., 2007; Sinsch, 2014; Smith & Green, 2005; Trochet et al., 2017). Consequently, we calculated the percentage of three land cover types: urban, agricultural, and natural within a 1‐km radius of the capture site using QGIS (version 3.10.13). The areas corresponding to different land cover types were extracted from the CORINE Land Cover geographic database using level ‘one’ of the nomenclature. Depending on the percentage of each type of land cover, the sites were classified into four categories of habitats (Table 2). Sites with more than 80% of urban or natural land cover were classified as such. Sites with mixed percentages of natural and agricultural habitat were classified as agricultural because the percentage of agricultural land was almost equal to the percentage of natural land, making them very different from natural habitats. Sites without a dominant land type were classified as mixed. GPS coordinates of each site were recorded using a Garmin GPSMAP 64st (Garmin, Olathe, Kansas).

**TABLE 1:** Sampled sites and their habitat classification and macrohabitat measurements.

Location	*N*	E	Classification	Surface (m^2^)	Water temperature (°C)—May—	pH	O_2_	Turbidity (FNU)	Conductivity	Air temperature (°C)—March —	Forest cover (%)	Aquatic plant cover (%)
Auffargis	48.69017	1.91673	Natural	14,086						12.10	20	10
Fontenailles	48.52185	2.89927	Natural	400	24.05	6.68	9.8	63.0	223	13.30	10	90
Poigny‐la‐forêt	48.68539	1.74443	Natural	13,046	11.93	6.37	60.2	14.5	81	10.1	10	40
Recloses	48.34608	2.66032	Natural	127	13.04	6.76	29	37.5	140	15.80	35	90
Chamarande	48.52026	2.22531	Agricultural		13.41	6.18	10.7	116.9	93		5	85
Chenoise‐Cucharmoy	48.62991	3.20974	Agricultural	699	25.03	8.14	43	20.2	500	13.50	12.5	25
Les Bréviaires	48.71821	1.82632	Agricultural	48,330	11.18	7.26	46.3	416.0	205	9.50	0	100
Marines	49.15710	1.99530	Agricultural	608						14	80	
Ury	48.35085	2.60803	Agricultural		14.49	7.11	7.4	693.0	296	13.00	15	80
Vieille‐Eglise	48.66147	1.88003	Agricultural	1056	10.97	6.64	15.3	40.2	356	5.60	0	99
St Rémy lès Chevreuses 1	48.712300	2.106115	Mixed	707	18.67	6.89	15.2	206.0	967	12.00	0	20
St Rémy lès Chevreuses 2	48.710212	2.100321	Mixed	170	26.39	9.09	54.5	16.5	426	12.00	0	100
Saint‐Fargeau‐Ponthierry	48.56036	2.52374	Mixed	303	21.10	7.53	32	144.0	88	11.00	33	25
Paris—Bois de Vincennes	48.83488	2.43646	Urban	598	21.27	6.11	120.4	7.4	364	10.10	100	30
Paris—Jardin Naturel Pierre Emmanuel	48.85892	2.39968	Urban	100		7.37		5.9	827	12.90	10	30

**TABLE 2:** Site classification according to the percentage of land types extracted from Corine Land Cover over a radius of 1 km around the sampling point.

Habitat type	Artificial	Agricultural	Natural
Urban	>80%	<20%	<20%
Mixed	>20%	>20%	>20%
Agricultural	<20%	>20%	>20%
Natural	<20%	<20%	>80%

The microhabitat at each site was characterised by several measurements. The surface of the ponds was measured in Image J (Schneider et al., 2012) using satellite images from Google Maps (2023), Geoportal (2021), and the QGIS ‘openstreetmap’ plugin (2023) depending on which source gave the best visibility of the pond. Water and air temperatures were recorded using a digital K‐type thermocouple (TFC‐305P, OneTemp, Marleston, South Australia). The proportion of forest cover on site was defined as the percentage of the pond surface covered by trees and shrubs. The proportion of the pond covered by aquatic vegetation was also recorded. A multiparameter probe (HI 9829, Hanna instruments, Lingolsheim, France) was used to measure the water temperature, pH, dissolved oxygen, turbidity, and conductivity for each pond.

### 2.3 Morphological measurements

**FIGURE 1:**
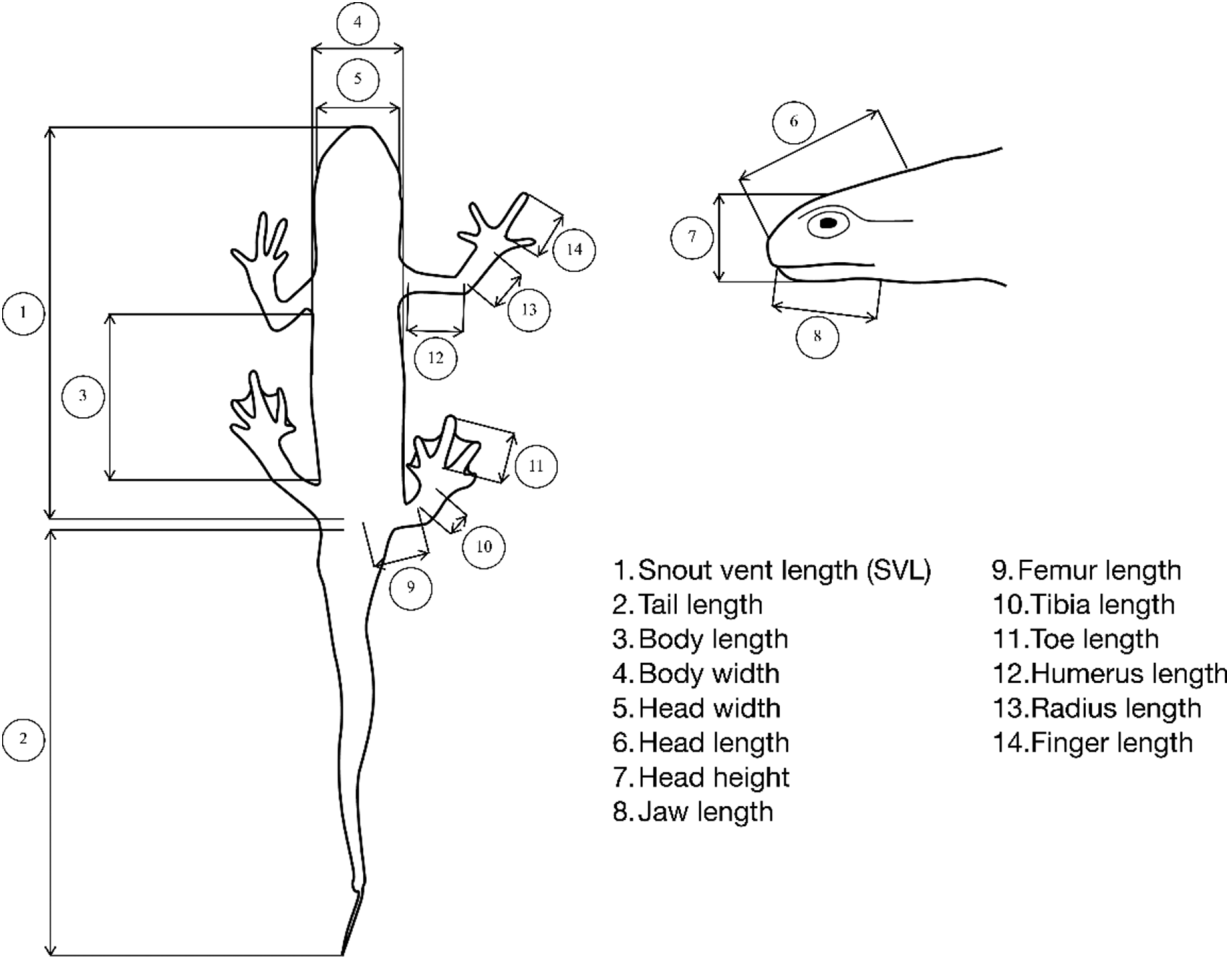
Morphological measurements taken on *Lissotriton helveticus*.

We captured a total of 167 palmate newts between March and May during their aquatic phase (Table 3). For each individual, we determined its sex and measured body mass and 14 morphological measurements (Figure 1). These measurements were taken directly in the field by a single operator (RF), after which the newts were released. All measurements were taken using digital callipers (Mitutoyo CD‐20DAX, ±0.01 mm).

**TABLE 3:** *Lissotriton helveticus* captured.

Localisation	Natural	Agricultural	Mixed	Urban	Total
Female	22	29	13	12	76
Male	20	28	21	22	91
Total	42	57	34	34	167

To test the repeatability of the measurements, morphological measurements were repeated ten times on three *Lissotriton helveticus* individuals of similar size. Using a multivariate analysis of variance (MANOVA), we verified that measurement bias was negligible as all individuals were significantly different from one another (*F* = 30.76, *p* < .001). Each variable was also individually examined using an analysis of variance (ANOVA), and all variables showed significant differences among individuals suggesting that our measurements were precise.

### 2.4 Captive maintenance

Sixteen individuals (8 females and 8 males) per habitat type (urban, mixed, agricultural, and natural) were captured (permit numbers 2022 DRIEAT‐IF/028‐035 and 2023 DRIEAT‐IF/031‐038) and transferred to the function and evolution laboratory at the National Museum of Natural History in Paris. Newts were housed by two in clear plastic containers (40 × 20 × 15 cm) with 5 cm of dechlorinated tap water and a stone allowing animals to leave the water. On a weekly basis, newts were fed with bloodworms and enclosures were cleaned twice a week. Animals were maintained on a 12‐h light–dark cycle at a temperature of 19°C. These animals were used to quantify locomotor performance and inflammatory responses. All individuals were acclimated for at least 5 days and were given 1 week of rest between different experiments (performance measurements and inflammatory responses). At the end of the experiments, all newts were released at their site of capture.

### 2.5 Locomotor performance

Animals were tested in an open rectangular plexiglass tank measuring 96 cm in length, 19 cm in width, and 38 cm in height. The bottom of the tank was covered with cork to provide traction during running. For swimming, the tank was filled with water to a depth of 8 cm at room temperature. We recorded locomotion using a high‐speed camera (Phantom Miro R311). The frame rate was set to 500 fps for running and 700 fps for swimming. We stimulated animals to move by lightly touching their tails. Each individual performed three trials. All the animals were measured during the same half‐day for each test. They were fed 2 days before and immediately after testing.

All videos were analysed using the ProAnalyst software (version 1.6.6.0; Xcitex Woburn, MA). We digitised the snout‐tip and exported the X and Y coordinates to Microsoft Excel. From the raw coordinates, we calculated the cumulative displacement profile. We then smoothed the displacement profile using a Butterworth filter implemented in Microsoft Excel and calculated maximum instantaneous speed and acceleration by numerical derivation. The highest speed and acceleration across the three trials were retained for further analysis.

### 2.6 Inflammatory responses

The inflammatory response was evaluated by measuring the swelling following the injection of phytohemagglutinin (PHA). PHA causes blood cells to clump together, leading to an inflammatory reaction. The thickness of the swelling is a reliable measure of immunocompetence and reflects the innate and adaptive immunity of individuals (Josserand et al., 2015) with a greater swelling indicating a more virulent inflammatory response. This test has been used and validated in numerous studies, including amphibians (Brown et al., 2011; Clulow et al., 2015; Murillo‐Rincón et al., 2017).

The palmate newts were injected with a PHA solution (0.01 mg/mL) dissolved in phosphate‐buffered saline (PBS) at the base of the hind limb. The other hind limb received an injection of 0.01 mL of PBS as a control. The choice of the limb (left or right) for the PHA solution injection was random and the measurer (RF) was blind to which limb was injected to avoid any bias or prior knowledge regarding the possibility of swelling. We measured the thickness of the base of the hind limbs using a calliper (Mitutoyo CD‐20DAX, ±0.01 mm), 1 h before the injection and at 12, 24, 36, and 48 h after the injection to monitor the evolution of the swelling. We repeated each measurement three times, and we used the median for statistical analysis.

To quantify the measurement error, we measured both hind limbs of one individual ten times each and estimated the standard error (SE) which was of 0.045 mm. Differences greater than this value can thus be attributed to a biological response.

### 2.7 Statistical analyses

All statistical tests were performed using RStudio version 4.0.4. (R Development Core Team, 2021) and IBM SPSS V29.

### 2.8 Habitat characterisation

A MANOVA was used to test the effect of habitat type on the following variables: air temperature, surface area of the pond, and forest cover. A second MANOVA was carried out to test the variables associated with the water body in the function of habitat type: water temperature, turbidity, conductivity, dissolved oxygen, pH, and aquatic plant cover. We subsequently tested for differences in turbidity using an ANOVA coupled with a post‐hoc Tukey test. We tested this hypothesis specifically as visual differences in turbidity were observed in the field.

### 2.9 Impact of the habitat type on morphology

We performed an ANOVA on Log_10_‐transformed snout‐vent length (SVL) to test whether newts captured in different habitats were different in size. As habitat significantly influenced SVL of females (*F*_3,72_ = 4.64, *p* = .005) and males (*F*_3,87_ = 3.62, *p* = .016), we used SVL as a covariate in all subsequent analyses. A multivariate analysis of covariance (MANCOVA), with SVL as a covariate, was used to test for differences in morphology among habitat types. We then conducted analyses of covariance (ANCOVA), with SVL as a covariate to test which variables differed. General Linear Hypothesis Tests (GLHT), using the “multcomp” package, were performed to determine the differences among the four habitats only for variables that showed statistical significance according to the ANCOVA.

To quantify residual body mass, a linear regression of Log_10_‐transformed body mass on Log_10_‐transformed snout‐vent length was run for each sex separately. Unstandardised residuals were extracted and used as a proxy for body condition (i.e. the mass of the muscles plus fat reserves irrespective of variation in snout‐vent length). An ANOVA on these residuals was conducted to test the effect of habitat on body condition. A post‐hoc Tukey test was used to test which habitat types differed from one another.

### 2.10 Impact of habitat type on locomotor performance

We conducted preliminary ANOVAs to check the effect of sex on maximum speed (running: *F*_1,62_ = 0.32, *p* = .57; swimming: *F*_1,60_ = 4.30, *p* = .042) and maximum acceleration (running: *F*_1,62_ = 0.75, *p* = .39; swimming: *F*_1,60_ = 2.93, *p* = .092). SVL had no effect on maximum running speed (*p* = .078) and acceleration (*p* = .055) nor on swimming speed (*p* = .80) and acceleration (*p* = .14). Consequently, ANOVAs were performed to test for differences in locomotor performance among habitats coupled to Tukey post‐hoc tests when significant differences were detected.

### 2.11 Morphological determinants of locomotor performance

To better understand which morphological traits drive variation in locomotor performance we ran backward regression models with Log_10_‐transformed limb and body dimensions as predictor variables and each of the four locomotor performance traits as our response variables. For the best models determined by the AIC criterion, we calculated the standardised partial regression coefficients (β) to evaluate the contribution of each variable to the final model.

### 2.12 Impact of habitat type on inflammatory responses

We first tested whether snout‐vent length impacted the swelling response, which was the case (*R* ^2^ = 0.42; *p* < .001). A two‐factor repeated measures ANOVA (using the “ez” package) was conducted to study the impact of different treatments (PBS and PHA) on the thickness of the hind limb over time. A repeated measures ANOVA was further used to test for differences between the two treatments. The *p*‐values of the tests were adjusted using a Bonferroni correction for multiple testing. Finally, we regressed the Log_10_‐transformed swelling response on Log_10_‐transformed snout‐vent length and extracted the unstandardised residuals. These were then used in a two‐way ANOVA to test for differences in the inflammatory response between sexes and habitat types.

## 3 RESULTS

### 3.1 Habitat characterisation

The microhabitat characteristics, i.e. air temperature, surface area of ponds, and forest cover, showed no difference according to the habitat type (Wilks' lambda = 0.37; *F*_9,14.75_ = 0.84; *p* = .59). The same was true for water characteristics (Wilks' lambda = 0.044; *F*_18,8.97_ = 1.00; *p* = .52). However, a tendency for differences in turbidity among habitat types was observed (*F*_3,9_ = 2.89, *p* = .09). The turbidity of water in urban habitats was lowest (x̄_urb_ = 6.65 FNU) followed by natural (x̄_nat_ = 38,34 FNU), mixed (x̄_mix_ = 171.67 FNU), and agricultural (x̄_agr_ = 257,25 FNU) habitats (Table 4).

**TABLE 4:** Tukey test results for turbidity based on habitats (bold = significant).

	Turbidity	
	*p*‐Value	Diff
Urban		
~Natural	.655	−1.149
~Agricultural	**.002**	**−3.710**
~Mixed	**<.001**	**−3.940**
Agricultural		
~Natural	**.012**	**−3.117**
~Mixed	.916	0.644
Natural		
~Mixed	**.005**	**−3.386**

### 3.2 Morphology

**FIGURE 2:**
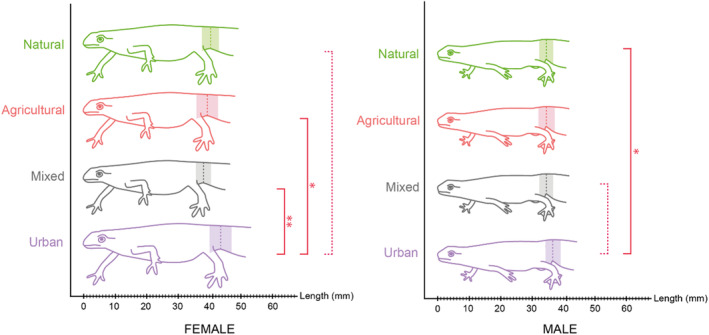
Effect of habitat on the snout‐vent length of female and male *Lissotriton helveticus* in four types of habitats: Natural habitat (green, *N*_female_ = 22; *N*_male_ = 20), agricultural habitat (orange, *N*_female_ = 29; *N*_male_ = 28), mixed habitat (grey, *N*_female_ = 13; *N*_male_ = 21), and urban habitat (purple, *N*_female_ = 12; *N*_male_ = 22). Red dotted line = trend, red full line = significant: *p ≤ .05; **p ≤ .01.

Habitat type had a significant influence on the morphology of females (MANCOVA: *Df* = 3,68; *Pillai* = 0.805, *p* = .040) and males (MANCOVA: *Df* = 3,86; *Pillai* = 0.861, *p* < .001) *Lissotriton helveticus*. In the urban habitat, individuals had a significantly greater SVL compared to other habitat types (Figure 2; Table 5).

**TABLE 5:** Tukey test results for SVL based on habitats (underlined = trend, bold = significant).

SVL	Female		Male	
	*p*‐Value	Diff	*p*‐Value	Diff
Urban				
~Natural	.092	2.597	**.012**	**2.261**
~Agricultural	**.026**	**3.017**	.189	1.342
~Mixed	**.002**	**4.550**	.054	1.840
Agricultural				
~Natural	.962	−0.420	.535	0.920
~Mixed	.440	1.533	.880	0.498
Natural				
~Mixed	.268	1.953	.938	−0.421

**FIGURE 3:**
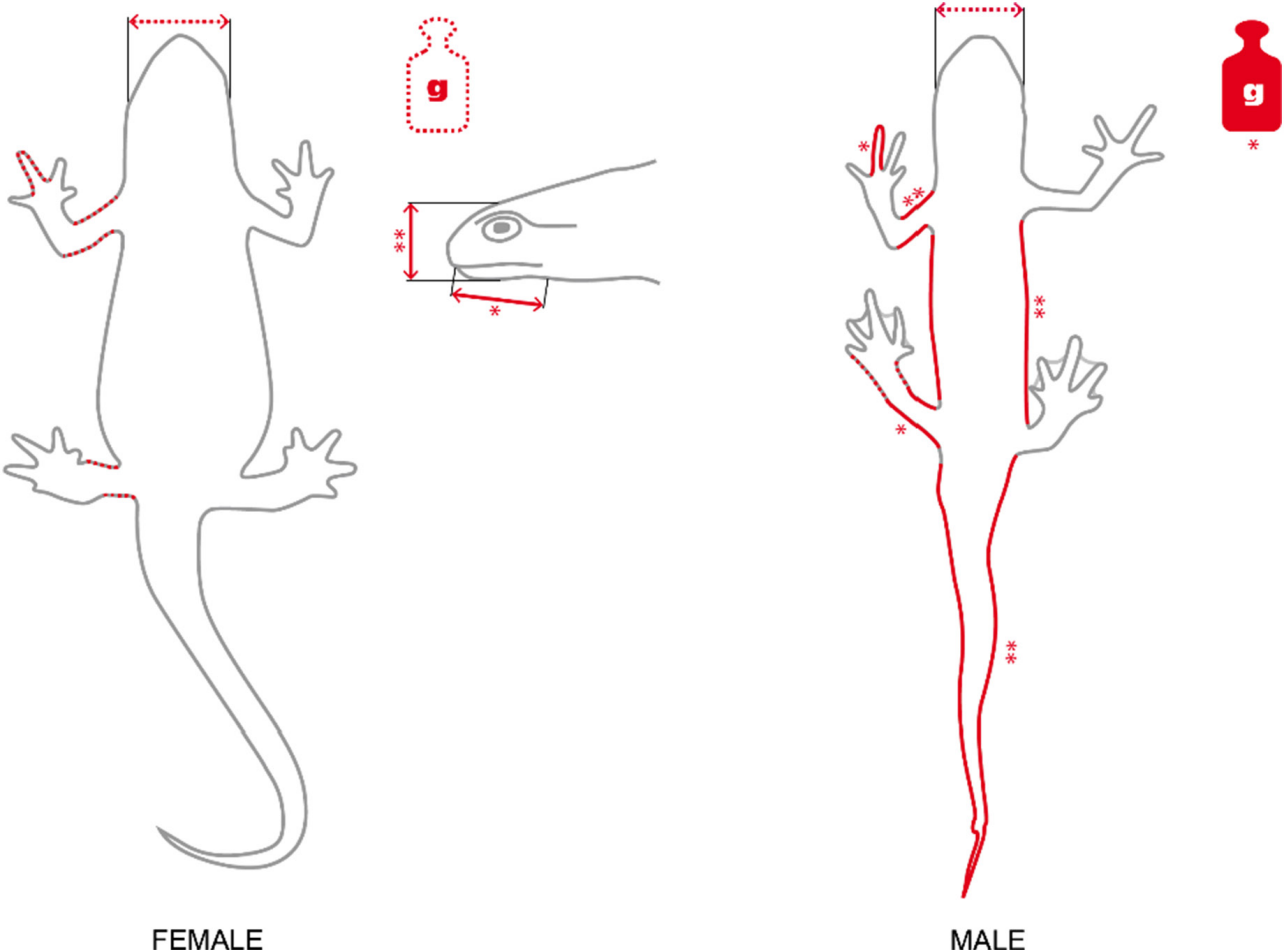
Effects of habitat type on morphology of female and male *Lissotriton helveticus*. Grey line = no effect, red dotted line = trend: 0.05 < *p* < .10, red full line = significant: *p ≤ .05, **p ≤ .01.

In addition to variation in snout‐vent length, the mass, tail length, body length, femur length, humerus length, and finger length for males and head height and jaw length for females showed significant differences among habitat types irrespective of variation in SVL. Additionally, there is a tendency for males to differ in head width and body length depending on the habitat type. For females, mass, head width, femur length, humerus length, and finger length tended to differ according to habitat type (Figure 3, Table 6).

**TABLE 6:** *p*‐Values of ANCOVA for morphological variables based on habitat type, accounting for SVL for both sexes of *Lissotriton helveticus* (underlined = trend, bold = significant).

Measures	Female		Male	
	*p*‐Value	Mean	*p*‐Value	Mean
Weight	.077	2.483	**.037**	**1.636**
Body length	.816	19.823	**.002**	**17.364**
Body width	.117	10.462	.939	7.286
Head length	.359	8.585	.657	8.109
Head width	.088	7.572	.091	6.818
Head height	**.012**	**4.551**	.197	4.159
Jaw length	**.020**	**8.544**	.197	8.086
Humerus length	.073	6.837	**.003**	**6.728**
Radius length	.493	4.589	.770	4.529
Finger length	.070	3.851	**.042**	**4.365**
Femur length	.087	6.240	**.033**	**5.874**
Tibia length	.268	3.681	.077	3.534
Toe length	.264	3.671	.152	4.206
Tail length	.793	35.278	**.002**	**33.011**

In urban habitats, both male and female newts were larger. For females, relative head height and relative jaw length were greater in the natural habitat (x̄_hea_ = 4.67 mm; x̄_jaw_ = 8.70 mm) compared to the agricultural habitat (x̄_hea_ = 4.31 mm; x̄_jaw_ = 8.26 mm; Figure 3, Table 7). In males, relative body mass was higher in urban habitats compared to natural habitats (x̄_nat_ = 1.40 g; x̄_urb_ = 1.80 g). The femur was relatively longer in the mixed habitat compared to agricultural and natural habitats (x̄_mix_ = 6.05 mm; x̄_agr_ = 5.70 mm; x̄_nat_ = 5.60 mm). Moreover, animals in mixed habitats had relatively longer fingers than those in urban habitats (x̄_mix_ = 4.49 mm; x̄_urb_ = 4.32 mm). The humerus was relatively shorter in animals from natural compared to urban habitats (x̄_nat_ = 6.15 mm; x̄_urb_ = 7.19 mm) for males. However, the relative body length of males was greater in the urban habitat (x̄_urb_ = 18.62 mm; x̄_mix_ = 16.59 mm; x̄_nat_ = 16.51 mm). Furthermore, relative tail length differs among certain habitats, being relatively longer in the agricultural habitat compared to urban and natural habitats and relatively shorter in the urban habitat compared to the mixed habitat (x̄_agr_ = 34.04 mm; x̄_mix_ = 33.19 mm; x̄_urb_ = 32.60 mm; x̄_nat_ = 31.22 mm). For both sexes, a tendency for a greater head width in urban habitats compared to agricultural habitats was observed (Female: x̄_urb_ = 8.10 mm; x̄_agr_ = 7.38 mm; Male: x̄_urb_ = 7.11 mm; x̄_agr_ = 6.69 mm; Table 7). Habitat type also had an influence on the ‘body condition’ of males (*F*_3,87_ = 3.37; *p* = .02) but not females (*F*_3,69_ = 1.57; *p* = .20). For males, the body condition was lower in natural habitats compared to agricultural and urban habitats (Table 8).

**TABLE 7:** *p*‐Values of GLHT for morphological variables based on environments, accounting for SVL covariate, in both sexes of *Lissotriton helveticus* (underlined = trend, bold = significant).

Female						Male					
Measures	x̄_urb_ ± se	x̄_mix_ ± se	x̄_agr_ ± se	x̄_nat_ ± se	*p*‐Value	Measures	x̄_urb_ ± se	x̄_mix_ ± se	x̄_agri_ ± se	x̄_nat_ ± se	*p*‐Value
Weight	2.977 ± 0.461	2.137 ± 0.299	2.391 ± 0.451	2.421 ± 0.317	P_urb~agr_ = .094 P_urb~mix_ = .069	Weight	1.815 ± 0.384	1.615 ± 0.240	1.662 ± 0.301	1.412 ± 0.241	**P**_**urb~nat**_ **= .047** P_agr~nat_ = .077
Head width	8.137 ± 0.439	7.340 ± 0.271	7.411 ± 0.368	7.584 ± 0.523	P_urb~agr_ = .054	Head width	4.365 ± 0.694	6.794 ± 0.406	6.715 ± 0.664	6.623 ± 0.950	P_urb~agr_ = .085
Humerus length	7.398 ± 0.752	6.681 ± 0.465	6.868 ± 0.566	6.558 ± 0.815	P_urb~nat_ = .073	Humerus length	7.216 ± 0.665	6.717 ± 0.556	6.735 ± 0.501	6.158 ± 0.868	**P**_**urb~nat**_ **= .001** P_agr~nat_ = .065 P_nat~mix_ = .050
Finger length	4.064 ± 0.444	3.944 ± 0.179	3.785 ± 0.526	3.761 ± 0.454	P_agr~mix_ = .087 P_nat~mix_ = .060	Finger length	4.326 ± 0.460	4.491 ± 0.336	4.456 ± 0.661	4.149 ± 0.547	P_urb~mix_ = .060
Femur length	6.581 ± 0.413	6.390 ± 0.307	6.119 ± 0.603	6.114 ± 0.789	x	Femur length	6.120 ± 0.672	6.058 ± 0.371	5.716 ± 0.462	5.614 ± 0.750	P_agr~nat_ = .063 P_nat~mix_ = .084
Head height	4.820 ± 0.439	4.558 ± 0.270	4.330 ± 0.368	4.679 ± 0.554	**P**_**agr~nat**_ **= .024**	Tibia length	3.774 ± 0.399	3.527 ± 0.300	3.491 ± 0.334	3.322 ± 0.425	P_urb~nat_ = .074
Jaw length	8.928 ± 0.685	8.449 ± 0.518	8.280 ± 0.446	8.719 ± 0.581	**P**_**agr~nat**_ **= .021**	Body length	18.661 ± 1.471	16.648 ± 1.112	17.389 ± 1.576	16.565 ± 1.392	**P**_**urb~nat**_ **= .009** P_urb~agri_ = .090 **P**_**urb~mix**_ **= .003**
						Tail length	32.753 ± 3.481	33.281 ± 2.436	34.209 ± 3.448	31.305 ± 2.997	**P**_**urb~agri**_ **= .005** **P**_**urb~mix**_ **= .046** **P**_**agri~nat**_ **= .042**

**TABLE 8:** *p*‐Values of Tukey test for male and female body condition by habitat.

	Mixed	Agricultural	Natural
Urban	P_M_ = 1.00	P_M_ = 0.99	**P**_**M**_ **= 0.049**
	P_F_ = 0.21	P_F_ = 0.25	P_F_ = 0.34
Mixed		P_M_ = 1.00	**P**_**M**_ **= 0.046**
		P_F_ = 0.97	P_F_ = 0.97
Agricultural			**P**_**M**_ **= 0.045**
			P_F_ = 1.00

*Note*: Bolded values are significant.

Abbreviations: F, female; M, male.

### 3.3 Locomotor performance

**FIGURE 4:**
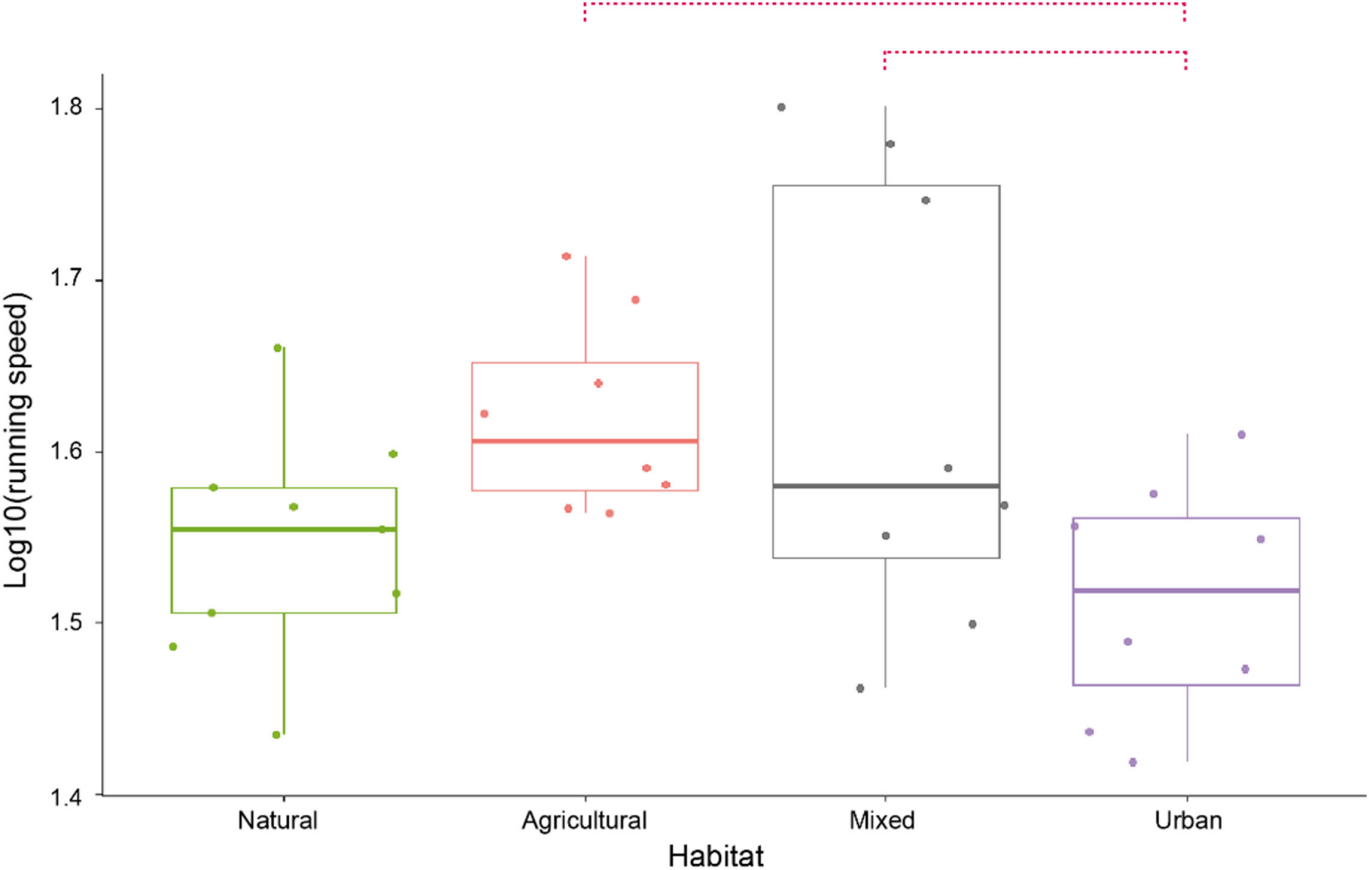
Running speed of female *Lissotriton helveticus* in four types of habitats: natural habitat (green, *N* = 9), agricultural habitat (orange, *N* = 8), mixed habitat (grey, *N* = 8), and urban habitat (purple, *N* = 8). Red dotted line = trend.

The MANOVA performed on running and swimming performance for females was not significant (Wilks' lambda = 0.57; *F*_12,66.43_ = 1.33; *p* = .23). However, the univariate ANOVA detected significant differences in running speed (*F*_3,28_ = 3.27; *p* = .036), with urban newts showing a tendency to be slower than newts from mixed (*p* = .073) and agricultural (*p* = .09) habitats (Figure 4; Table 9). For males, the MANOVA was also not significant (Wilks' lambda = 0.50; *F*_12,61.14_ = 1.52; *p* = .14). Moreover, none of the univariate ANOVAs showed differences among habitat types (all *p* > .05).

**TABLE 9:** *p*‐Values of a post‐hoc Tukey test testing for differences in maximum running speed in female *Lissotriton helveticus* (underlined = trend).

	Mixed	Agricultural	Natural
Urban	0.073	0.090	0.90
Mixed		1.00	0.27
Agricultural			0.31

*Note*: Also indicated are marginal means.

Our backward regression model retained a significant model for running speed (*R* ^2^ = 0.203; *p* = .009) with body mass, snout‐vent length, radius length, and tail length as predictors. Partial regression coefficients showed that whereas body mass and radius length positively impacted running speed (body mass: β = .44; radius length: β = .26), snout‐vent length and tail length did so negatively (snout‐vent length: β = −.53; tail length: β = −.30). For terrestrial acceleration a significant model was also retained (*R* ^2^ = .238 *p* = .003) with body mass (β = .42) and tibia length (β = .28) as positive predictors and snout‐vent length (β = −.51) and femur length (β = .37) as negative predictors. For swimming speed, a significant model (*R* ^2^ = .29 *p* < .001) with tibia length as positive (β = .33) and the length of the longest toe (β = −.35) and humerus length (β = −.33) as negative predictors was retained. Finally, for swimming acceleration a significant model (*R* ^2^ = .23 *p* = .002) with tibia length (β = .26) and body length (β = .32) as positive and humerus length as negative predictor (β = −.37) was retained.

### 3.4 Inflammatory response

Whereas the thickness of the control hind limb (PBS) showed no significant variation over time (*F*_3,86_ = 0.43; *p* = 1.0), the limb that received the PHA injection exhibited significant changes (*F*_3,86_ = 10.2; *p* < .001). A statistically significant interaction between treatment and post‐injection time was observed (*F*_3,86_ = 4.61; *p* = .049). An effect of treatment was demonstrated at 24 h post‐injection with an increase in thigh width (x̄_0h_ = 2.40 mm; x̄_24h_ = 2.44 mm; *Df* = 1, *F* = 21.5, *p* < .001), but not at other time points. Consequently, we examined the differences in thickness between the PHA‐injected limb and the control limb at 24 h post‐injection and tested for differences between habitat type and sex. Whereas the effect of habitat type (*F*_3,51_ = 0.94; *p* = .43) and the interaction between habitat type and sex (*F*_3,51_ = 1.63; *p* = .19) were non‐significant, sex differences were (*F*_1,51_ = 19.84; *p* < .001) with females showing a stronger inflammatory response than males across all habitat types.

## 4 DISCUSSION

Understanding responses of amphibians to human‐modified habitats is fundamental to the conservation of species and habitats as changes in land use require species to adapt. Population response studies make it possible to predict future trends and thus to establish future conservation priorities (Donihue & Lambert, 2015; Lambert et al., 2021). The present study builds on previous studies examining the effect of land use differences on newt morphology (Trochet, Le Chevalier, et al., 2016) but adds integrative measures (i.e. performance and immunity) of the effects of land use differences on newt populations. Our results show that newt morphology was impacted by habitat type. However, the impact on locomotor performance and inflammatory responses remained moderate. For males, individuals from the urban habitat show a greater body size while the smallest newts were found in the natural habitat. Interestingly, females from agricultural and mixed habitats tended to have a higher running speed and females overall showed a stronger inflammatory response than males across all habitats.

### 4.1 Sex‐specific characteristics

Males and females exhibited morphological differences irrespective of variation in body size according to the habitat they were caught in. Whereas these differences were significant for males, for females mostly tendencies were observed (Tables 6 and 7). This might partly be explained by the fact that our sample contained 15 fewer females than males (Table 3), lowering our ability to detect differences between sites. However, females did show significant differences in relative head height and jaw length among habitats. These variations in head dimensions might be driven by different diets in different habitat types. During the breeding season females move less than males (Bellis, 1968). This greater sedentary lifestyle could lead to dietary specialisation and make females more sensitive to variation in food availability. Dietary analyses through stomach flushing could allow us to test this hypothesis. Males showed body and tail length differences among habitats which could be driven by differences in the physical structure of the habitat (Trochet, Le Chevalier, et al., 2016; Winchell et al., 2016) or the effect of habitat type on secondary sexual characters and male attractiveness (De Solan et al., 2022).

The examination of the inflammatory response showed differences in the thickness of the limb before and after the injection of phytohaemagglutinin (PHA). The difference in swelling between the limb injected with phosphate‐buffered saline PBS (control) and PHA was strongest 24 h after injection which clearly demonstrates a reaction linked to the PHA. This observation is consistent with the literature (Clulow et al., 2015; Martin et al., 2006). Whereas there was no difference in the inflammatory response among habitats, females showed a stronger inflammatory response compared to males across all habitats. In other taxa like wall lizards (*Podarcis muralis*), it has been shown that macrophages in females are more active as compared with males because androgens have a suppressive effect on male macrophage activity (Mondal & Rai, 1999). This may explain the observed differences in the newts studied here as well.

### 4.2 Newts in urban versus natural habitats

In urban habitats, the body size of newts, for both sexes, was greater than in mixed, agricultural, or natural habitats. Other measures, such as head width in females and body mass, humerus length, and body length in males, were also found to be greater in this habitat. Several hypotheses can be put forward to explain these observations. First, the urban habitat is highly fragmented, forcing species to modify their spatial distribution (Ditchkoff et al., 2006). For instance, Löfvenhaft et al. (2004) observed that the distribution of amphibians over time is negatively related to increased fragmentation in the urban area of Stockholm. Indeed, amphibians generally show low mobility: 94% move over distances of less than 1 km (Phillips et al., 2007; Sinsch, 2014; Smith & Green, 2005) making them particularly sensitive to habitat modification. Fragmentation generates populations that are isolated into small areas (Forman, 1995). This may in turn reduce overall movement and energy consumption associated with locomotion. This energy could then potentially be allocated to growth, resulting in larger animals in more fragmented sites.

Alternatively, the observed size differences could be explained by a greater abundance of food, differences in longevity, or changes in prey availability in the urban habitat. Numerous comparative studies have been carried out on macroinvertebrate communities in urban and natural habitats. However, results are mixed, with some studies showing the same diversity in both habitat types (Hill et al., 2016), yet others showing differences (Meland et al., 2020). Mesopredator species diversity is generally lower in urban areas (Luck & Smallbone, 2010), and competition for resources with other species occupying the same niches is thus likely lower. This means that even if the food available in the two habitat types is the same, the quantity of food available per newt will be greater, all else being equal. The analysis of body condition did show higher relative body mass in males in urban habitats. Stomach flushing would be insightful to estimate the quantity and diversity of prey eaten (Fasola & Canova, 1992; Legler, 1977; Legler & Sullivan, 1979), in addition to studies of prey availability.

Predation pressure is also likely different in urban areas: natural predators, such as snakes, are rare whereas other predators, such as domestic cats and dogs, are present in large numbers (Koenig et al., 2002). However, these animals are, most of the time, unapt to predate on newts which live in the water or are inaccessible because of their secretive lifestyle. Birds are, however, present in cities, but most do not feed on newts. An inspection of the presence of birds that typically prey on newts (such as herons or kingfishers) using iNaturalist shows that these are mostly absent in the urban sites sampled here. With lower predation pressure, selection on locomotor capacity would likely be reduced, possibly resulting in larger and heavier individuals.

The urban heat island effect in cities is well documented (Battles & Kolbe, 2019; Oke, 1982) and could also impact growth rates. Indeed, as ectotherms, the development of amphibians is highly dependent on temperature (Zuo et al., 2012). A higher temperature in urban habitats could enable them to grow faster. Yet, our data did not show a difference in temperature among habitats, perhaps because this is a one‐off survey rather than an estimate of temperature variation over the duration of the entire aquatic phase. Finally, as amphibians grow continuously, older individuals are larger (Liao & Lu, 2012) suggesting that newts may live longer in urban habitats, consistent with the idea that predation is lower. A skeletochronological study would, however, be needed to determine the age of individuals (Peng et al., 2022). In summary, our results are consistent with lower energy expenditure in urban habitats due to restricted movement, an increase in food availability, a reduction in predation pressure and increased survival, and higher temperatures possibly favouring growth, all of which could contribute to the evolution of larger body size in urban habitats (Ditchkoff et al., 2006).

In contrast, individuals in the natural habitat were generally smaller than those captured in other habitats: body size and, for males specifically, relative body mass, humerus length, body length, and tail length were smaller. As limb size is linked to locomotion, smaller limbs could imply slower movements. Because of their limited mobility, the structural complexity of the habitat is important in affecting predation pressure and competitive interactions (Blouin‐Demers & Weatherhead, 2002; François et al., 2021). Habitat complexity in the natural habitat could lead individuals to favour hiding and crypsis rather than moving faster. Our locomotion performance results did not show marked differences in either running or swimming among habitat types. Yet, other types of locomotion such as endurance capacity could be affected by the shorter limbs observed in natural habitats. For females, head height and jaw length are greater in the natural habitat compared to the agricultural habitat. This suggests that female newts may be consuming larger prey in natural habitats. By specialising on larger prey, females may be able to avoid competition with males of their own species (Schoener, 1967, 1968), yet this remains to be investigated further.

### 4.3 Newts in mixed and agricultural habitats

The relative femur length, humerus length, finger length, and tail length of males were significantly impacted by the type of habitat and females showed similar trends. The femur length and finger length tend to be longer in mixed habitats, made up of 30% of all the other types of habitats. Longer limbs may help moving around in a diversity of habitats such as the mixed habitats sampled. This hypothesis could be linked to our locomotor performance data. Indeed, females from mixed and agricultural habitats tended to be faster than those living in natural and urban habitats. Yet, our regression models suggest that relatively longer humeri negatively impact most performance traits measured. This suggests that selection is likely not acting on performance traits like speed or acceleration but possibly other traits like endurance capacity or manoeuvrability (Vanhooydonck et al., 2000). Conversely, performance traits like speed or acceleration may be impacted more by muscle mass and cross‐sectional area than external limb dimensions (Vanhooydonck et al., 2006). The agricultural habitat is also a composite environment with 50% natural and 50% agricultural habitat. Overall, our data suggest that compound habitats lead to selection on limb dimensions and locomotor performance. These results are in line with a previous study which showed morphological selection depending on habitat type for the same species: newts captured close to roads have shorter legs suggesting that roads select for animals with lower mobility less likely to cross the roads (Trochet, Le Chevalier, et al., 2016). In our study males from mixed and agricultural habitats had longer tails than those from the urban and natural habitats. Although tail length could have an impact on swimming performance, this was not observed; rather tail length negatively impacted terrestrial running speed. Our data on pond surface area, air and water temperatures, water physical features, and vegetation cover also showed no differences among habitat types. However, the tail of males is an important secondary sexual feature that plays a part in female choice during mating. A relatively larger tail could constitute a more visible signal especially important in more turbid waters. In agricultural and mixed habitats, a tendency towards a higher turbidity was detected, which could explain the longer tails in these habitats.

## 5 CONCLUSION

This study demonstrates that land use differences result in changes in morphology in a newt. In urban habitats, newts were larger and heavier and had a greater body condition. This might be the result of lower mobility, a change in food availability, a lower predation pressure, increased longevity, or reduced competition. In natural habitats, newts are smaller, but some features such as female head size were greater suggesting dietary specialisation in females. In mixed and agricultural habitats newts have longer limbs, which might improve mobility in structurally complex habitats. The relatively longer tail length in males in urban habitats suggests a role of secondary sexual characters in more turbid waters. Future studies would benefit from investigating dietary variation, food availability, growth, and population dynamics to better understand how differences in land use impact the evolutionary trajectory of newts.

## AUTHOR CONTRIBUTIONS

**Soline Bettencourt‐Amarante:** Conceptualization (equal); data curation (lead); funding acquisition (lead); methodology (equal); supervision (equal); writing – original draft (lead); writing – review and editing (equal). **Robin Furet:** Data curation (equal); formal analysis (equal); writing – original draft (equal). **Raphaëlle Abensur:** Data curation (equal); formal analysis (equal). **Anthony Herrel:** Conceptualization (lead); formal analysis (equal); methodology (equal); supervision (equal); writing – review and editing (equal).

## CONFLICT OF INTEREST STATEMENT

The authors declare no conflicts of interest.

## Data Availability

The data are available at: https://doi.org/10.48579/PRO/397DWC.
